# SARS-CoV-2 Diagnostic Tests: Algorithm and Field Evaluation From the Near Patient Testing to the Automated Diagnostic Platform

**DOI:** 10.3389/fmed.2021.650581

**Published:** 2021-04-06

**Authors:** Nicolas Yin, Cyril Debuysschere, Marc Decroly, Fatima-Zohra Bouazza, Vincent Collot, Charlotte Martin, Fanny Ponthieux, Hafid Dahma, Marius Gilbert, Magali Wautier, Cecile Duterme, Nathalie De Vos, Marie-Luce Delforge, Stefano Malinverni, Frédéric Cotton, Magali Bartiaux, Marie Hallin

**Affiliations:** ^1^Department of Microbiology, LHUB-ULB, Université Libre de Bruxelles, Brussels, Belgium; ^2^General Practitioner, Brussels, Belgium; ^3^Emergency Department, Centre Hospitalier Universitaire Saint-Pierre, Université Libre de Bruxelles, Brussels, Belgium; ^4^Department of Infectious Diseases, Centre Hospitalier Universitaire Saint-Pierre, Université Libre de Bruxelles, Brussels, Belgium; ^5^Department of Clinical Chemistry, LHUB-ULB, Université Libre de Bruxelles, Brussels, Belgium; ^6^Spatial Epidemiology Laboratory, Université Libre de Bruxelles, Brussels, Belgium; ^7^Institut de Biologie Clinique, Université Libre de Bruxelles, Brussels, Belgium; ^8^Center for Environmental Health and Occupational Health, Public Health School, Université Libre de Bruxelles, Brussels, Belgium

**Keywords:** COVID-19, SARS-CoV-2, immunoassay, diagnostic, antigen, PCR, point-of-care, NAAT

## Abstract

**Introduction:** Since the first wave of COVID-19 in Europe, new diagnostic tools using antigen detection and rapid molecular techniques have been developed. Our objective was to elaborate a diagnostic algorithm combining antigen rapid diagnostic tests, automated antigen dosing and rapid molecular tests and to assess its performance under routine conditions.

**Methods:** An analytical performance evaluation of four antigen rapid tests, one automated antigen dosing and one molecular point-of-care test was performed on samples sent to our laboratory for a SARS-CoV-2 reverse transcription PCR. We then established a diagnostic algorithm by approaching median viral loads in target populations and evaluated the limit of detection of each test using the PCR cycle threshold values. A field performance evaluation including a clinical validation and a user-friendliness assessment was then conducted on the antigen rapid tests in point-of-care settings (general practitioners and emergency rooms) for outpatients who were symptomatic for <7 days. Automated antigen dosing was trialed for the screening of asymptomatic inpatients.

**Results:** Our diagnostic algorithm proposed to test recently symptomatic patients using rapid antigen tests, asymptomatic patients using automated tests, and patients requiring immediate admission using molecular point-of-care tests. Accordingly, the conventional reverse transcription PCR was kept as a second line tool. In this setting, antigen rapid tests yielded an overall sensitivity of 83.3% (not significantly different between the four assays) while the use of automated antigen dosing would have spared 93.5% of asymptomatic inpatient screening PCRs.

**Conclusion:** Using tests not considered the “gold standard” for COVID-19 diagnosis on well-defined target populations allowed for the optimization of their intrinsic performances, widening the scale of our testing arsenal while sparing molecular resources for more seriously ill patients.

## Introduction

At the time of writing (January 7, 2021), Belgium is emerging from a second wave of COVID-19 epidemic. The World Health Organization (WHO) recommended mass use of reverse transcription real-time PCR (RT-PCR) to detect active SARS-CoV-2 infections (1). However, the unprecedented high volume of samples reaching laboratories led to global scarcities of reagents and delays making prolonged containment measures less acceptable by the population (2). Since then, a new set of diagnostic tools have been developed, such as antigen detection immunoassays or molecular point-of-care tests. These tools could allow diversification of testing strategies and decrease shortages and overflows.

Thanks to their high sensitivity, ranging from 73.9 to 89.5% for high viral load samples [10^5^-10^7^ RNA copies/swab (3)], and their overall specificity (4, 5), antigen-detection rapid diagnostic tests have been integrated in several countries' testing strategies (6–10)^1^^,^^2^. Both Centers for Disease Control and Prevention (CDC) (11) WHO (12) and European Center for Disease Control and Prevention (ECDC) (13) have issued guidelines for their use. However, practical considerations are still lacking (including the best target populations). Meanwhile, several manufacturers have developed molecular point-of-care tests, most of which additionally target influenza and/or RSV (14, 15) while others offer wider respiratory syndromic panel (16).

In addition, high throughput antigen-dosing systems based on chemiluminescence enzyme immunoassay represent an interesting alternative (17). This solution, recently deployed in German airports, is a striking example of delocalized laboratory medicine (18).

Following this expansion of available diagnostic tools, a deeper reflection has come to light on the best use of these various testing solutions according to their sensitivity, their turnaround time, the context in which the result will be used (patient vs. population-centered approach), the kinetics of the epidemic and the availability of reagents and consumables (19).

All of the above may partly explain the apparent confusion we are currently witnessing in the deployment of antigen rapid diagnostic tests and/or molecular point-of-care tests in most industrialized countries, either in terms of choosing the most appropriate diagnostic tests or the target population to apply these tests to. We would like to share here the results of evaluations we performed on four antigen rapid diagnostic tests, one automated antigen dosing assay and one molecular point-of-care test for the diagnosis of COVID-19, not only from an analytical “laboratory” point-of-view but also through their field implementation during the second Belgian COVID-19 wave. Using different techniques at different levels in a multi-step, integrated, and adaptive diagnostic algorithm helped us to diversify and increase our overall testing capacity.

## Methods

### Population

LHUB-ULB (Laboratoire Hospitalier Universitaire de Bruxelles—Universitair Laboratorium Brussel) is a clinical laboratory serving five university hospitals (containing a capacity of around 3,000 beds) as well as a network of general practitioners in Brussels, Belgium. LHUB-ULB's service area covers 700,000 inhabitants (20). From July to September 2020, patients undergoing a SARS-CoV-2 RT-PCR were retrospectively categorized through a structured algorithm into four categories according to the information provided on the orders: symptomatic outpatients, hospital admissions (symptomatic or not), asymptomatic high-risk contacts, or mandatory screenings. The RT-PCR median C_T_ values from these four groups were compared using the Tukey-Kramer method.

### Symptomatic Cases Definition

We used the case definition provided by the Belgian national health institute (Sciensano) for COVID-19 (21). The acute apparition of one major symptom, the presence of two minor symptoms, or the aggravation of chronic respiratory symptoms without any other obvious cause was defined as a possible case (Supplementary Table 1). A confirmed case was a person with a SARS-CoV-2 positive sample.

### Diagnostic Tests

#### Antigen Rapid Diagnostic Tests

Four lateral-flow immunoassays were evaluated: Panbio™ COVID-19 Ag Rapid Test Device (Abbott Rapid Diagnostics, Germany), BD Veritor™ SARS-CoV-2 (Becton-Dickinson and Company, USA), COVID-19 Ag Respi-Strip (Coris BioConcept, Belgium) and SARS-CoV-2 Rapid Antigen Test (SD Biosensor, Republic of Korea). Reading was performed by trained operators except for the BD Veritor™ for which an automated reader (BD Veritor™ System) was used.

An analytical performance study was performed using nasopharyngeal swabs. The swabs preserved in universal transport media (UTM) were sent to our laboratory for a SARS-CoV-2 RT-PCR, and then kept refrigerated overnight after the RT-PCR was performed. The four assays were performed at the same time by two trained operators. The amount of UTM engaged was according to the recommendations by each manufacturer for evaluation purposes but not for clinical use.

After the performance study, antigen rapid diagnostic tests were done in point-of-care settings, either a practice within our network of general practitioners, or in the emergency room of the Saint-Pierre university hospital. Each possible COVID-19 outpatient, who was within 7 days of symptoms onset, was offered an antigen rapid diagnostic test and informed that a negative result would require an additional sampling for RT-PCR as recommended at the time (21). Each antigen rapid diagnostic test sampling and test procedure was performed according manufacturer instructions (Supplementary Table 2).

The user-friendliness of each antigen rapid diagnostic test was assessed with a four-part questionnaire adapted from the Scandinavian evaluation of laboratory equipment for point-of-care testing SKUP/2008/114 evaluation (22).

#### Molecular Point-of-Care Test

To assess the analytical performance of the Cobas® Liat SARS-CoV-2 & Influenza A/B nucleic acid test (Roche Molecular Systems, USA), nasopharyngeal swabs, which were sent to our laboratory for a SARS-CoV-2 RT-PCR and tested positive, were kept refrigerated overnight before testing. In addition, frozen samples from February 2020 which underwent at that time a Cobas® Liat Influenza A/B & RSV RT-PCR assay were also tested.

#### Automated Antigen Dosing Assay

Antigen dosing was performed using the Lumipulse® *G* SARS-CoV-2 Ag (Fujirebio, Japan) assay, expressing the dosage in pg/mL. For biosafety consideration, a viral-deactivation step (56°C heating for 30 min) was added to the manufacturer's instructions protocol (23).

The analytical performance study was performed on UTM swabs kept refrigerated overnight after a SARS-CoV-2 RT-PCR. All available positive samples were selected. Negative samples were randomly selected to obtain a positive/negative ratio around 2:1.

In the second part of the evaluation, we evaluated the Lumipulse® performance on UTM samples sent to our laboratory for SARS-CoV-2 RT-PCR for patients who required scheduled hospital admission, COVID-19 contacts, or for healthcare workers.

### Gold Standard and Statistical Analysis

Analytical performance study of antigen rapid diagnostic tests, molecular point-of-care test and automated antigen dosing were carried out on three different sets of samples.

SARS-CoV-2 RT-PCR was considered as the gold-standard. Except for some antigen rapid diagnostic tests, for which negative results were controlled by various other RT-PCR protocols, samples underwent the RealTi*m*e SARS-CoV-2 assay (Abbott Molecular, USA) on our *m*2000 platform. As detection of both targeted genes (RdRp and N) is performed using the same fluorophore, the C_T_ values of this assay are observed up to 32 cycles, and not comparable with C_T_ values of other RT-PCR assays. Consequently, only the C_T_ values obtained using the RealTi*m*e SARS-CoV-2 assay were considered.

Statistical analyses and receiver operating characteristic (ROC) curves were performed using Analyse-it® for Microsoft Excel v3.80.

## Results

### Trends of C_T_ Value in the Different Populations

**Figure 1:**
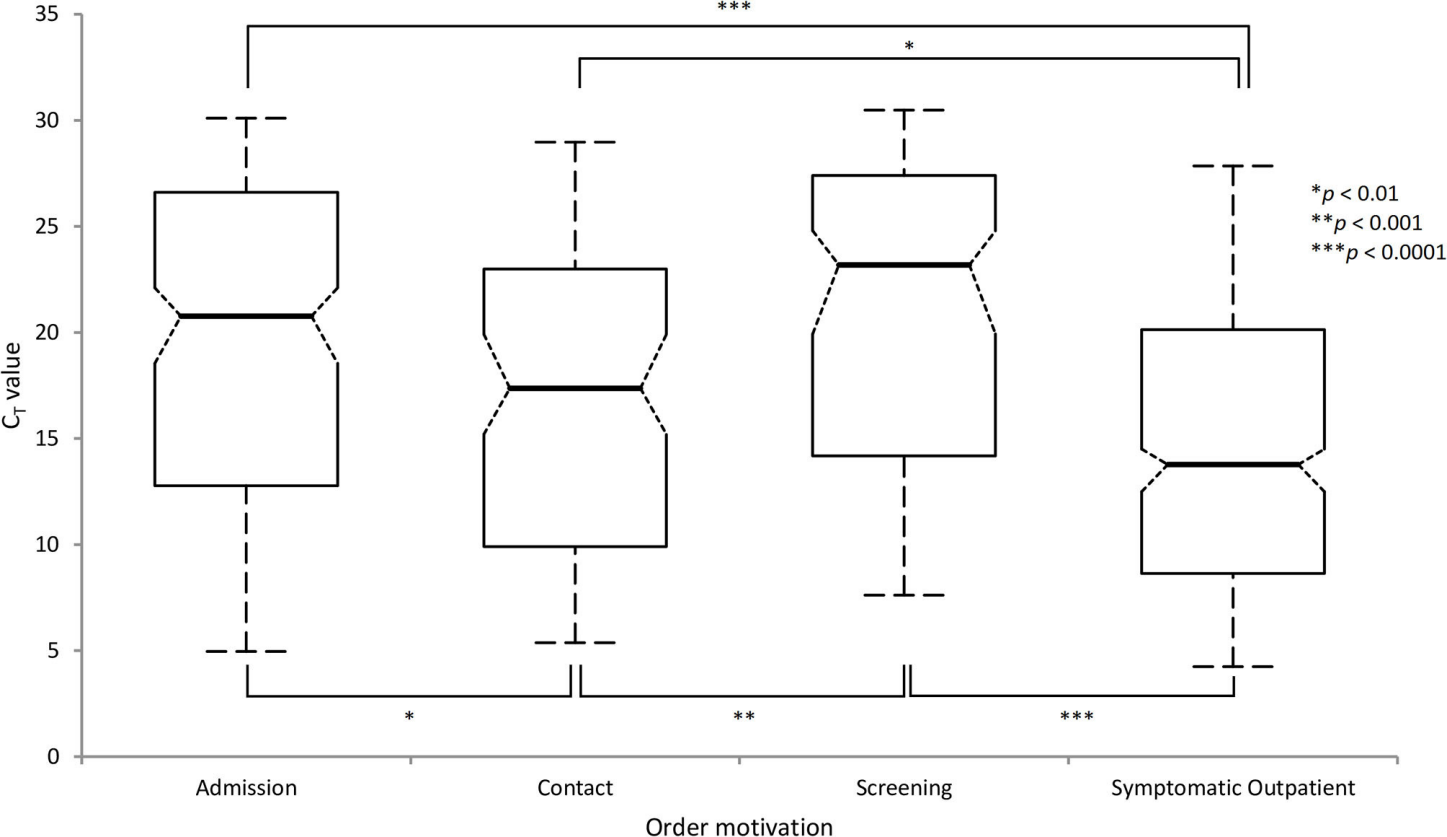
Distribution and comparison of C_T_ values in target populations according to the motivation of the order using Abbott RealTi*m*e SARS-CoV-2 solution (Comparisons using the Tukey-Kramer method).

LHUB-ULB performed 31,397 SARS-CoV-2 RT-PCR including 1,708 positive nasopharyngeal samples (5.4%) from 1,568 patients. 1,169 SARS-CoV-2 infected patients were categorized as follows: 580 symptomatic outpatients (49.6%), 318 admissions (27.2%), 178 contacts (15.2%), and 93 screenings (7.9%). The median C_T_ for symptomatic outpatients (13.8/32) was significantly lower than for any other group (Figure 1). The median C_T_ for contacts (17.4/32) was significantly lower than for admissions (20.8/32, *p* = 0.0044) and for screenings (23.2/32, *p* = 0.0002). Hence, antigen rapid diagnostic test was considered for symptomatic outpatients, automated antigen dosing for screenings and molecular point-of-care tests for hospital admissions.

### Analytical Performance Studies

#### Antigen Rapid Diagnostic Tests

Ninety-nine UTM samples including 61 positive (C_T_ ranging from 3.86/32 to 30.94/32) were selected. In this frame, the sensitivities of each antigen rapid diagnostic test were ranging from 36.1 to 49.2% (Table 1). The latest C_T_ detected antigen rapid diagnostic tests was 18.06/32. No false positive result was observed.

**Table 1:** Compared analytical performances of four SARS-CoV-2 antigen rapid diagnostic tests using 99 nasopharyngeal swabs preserved in universal transport media as proxy vs. Abbott RealTi*m*e SARS-CoV-2 assay.

	**Buffer dilution factor**	**Sensitivity (IC_**95**_)**	**Specificity**	**Last C_**T**_ detected**
Panbio™ COVID-19	1/2	45.9% (34.0–58.3%)	100%	18.06/32
Coris COVID-19 Ag Respi-Strip	1/2	39.3% (28.1–51.9%)	100%	13.31/32
SD Biosensor™ SARS-CoV-2	1/2	49.2% (37.1–61.4%)	100%	18.06/32
BD Veritor™ SARS-CoV-2	1/6	36.1% (25.2–48.8%)	100%	13.9/32

#### Molecular Point-of-Care Test

The agreement of the Cobas® Liat with the *m*2000 system for SARS-CoV-2 diagnostic was of 90.9% (50/55) for positive samples. C_T_ values correlation between instruments was good (*R*^2^ = 0.931). The Cobas® Liat yielded positive results for all positive samples presenting a C_T_ value below 27.29/32 and yielded positive results for samples with C_T_ of up to 29.11/32. Eighteen of the 19 frozen Influenza A positive samples and 5 of the 6 frozen influenza B positive samples yielded coherent positive results. Agreements for negative samples were of 100% for each parameter.

#### Automated Antigen Dosing Assay

Two hundred fourteen samples were selected including 136 positive samples. ROC curve analysis yielded an area under the curve (AUC) of 0.893±0.021 (Supplementary Figure 1). The highest Youden Index was at a threshold of 13.75 pg/mL (sensitivity 67.7%, specificity 97.1%). At a threshold set at 1.32 pg/mL [similar to a previous study (17) and to the manufacturer proposed cut-off at 1.34 pg/mL (24)], sensitivity was 78.9% and specificity of 73.9%. To exclude any false positive, the threshold had to be set at 20.27 pg/mL (sensitivity 63.9%). Finally, using a C_T_ <20/32 as a judgement criterion, the AUC of the ROC curve was 0.984 ± 0.007 (Supplementary Figure 2) with an optimal Youden index at a threshold of 20.27 pg/mL (sensitivity 87.4%, specificity 98.1%).

### Elaboration of the Diagnostic Algorithm

**Figure 2:**
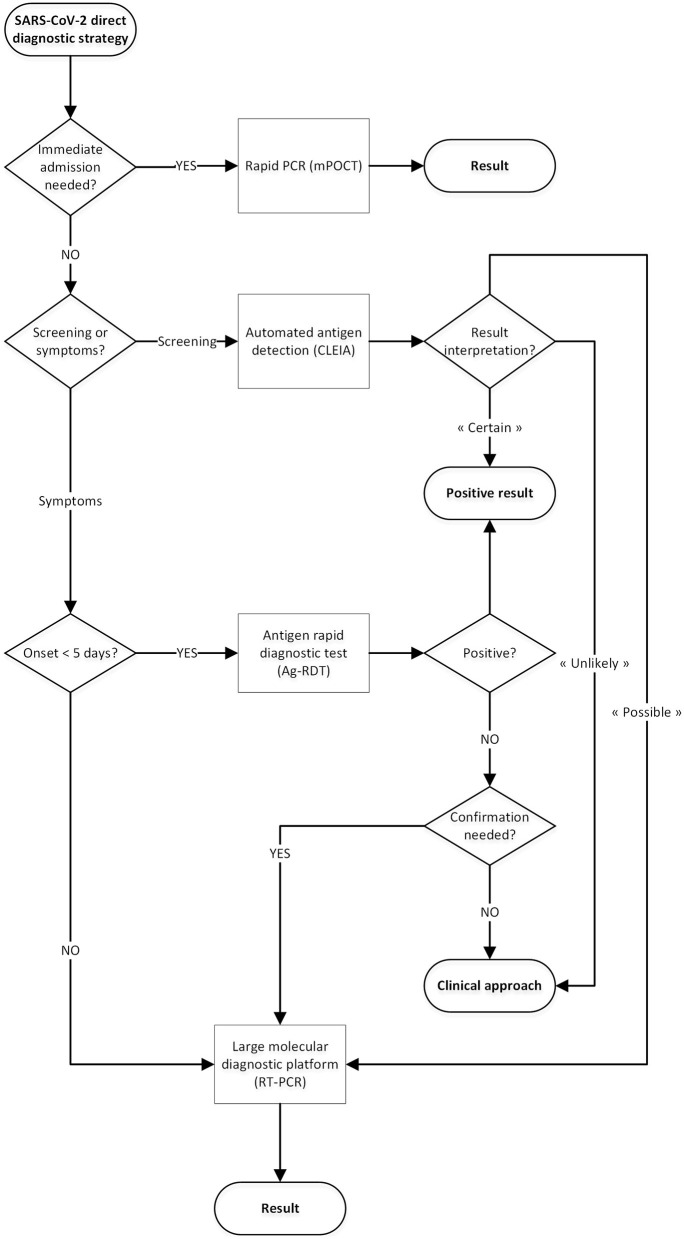
Proposal for a SARS-CoV-2 direct diagnostic decision algorithm.

Following these results, we elaborated the algorithm described in Figure 2: whereas the diagnosis of outpatients was mainly based on point-of-care antigen rapid diagnostic tests, the hospital algorithm combined antigen rapid diagnostic tests, molecular point-of-care tests and conventional RT-PCR in an integrative diagnostic strategy. Four clinical situations were further identified: screening of asymptomatic patients, patients requiring immediate admission, symptomatic outpatients with symptoms lasting for less or more than 5 days.

### Field Performance Evaluation

#### Antigen Rapid Diagnostic Tests

Four hundred ninety-four symptomatic outpatients underwent an antigen rapid diagnostic test. Two hundred and nine (42.3%) were positive. Sixteen negative antigen rapid diagnostic tests were excluded due to missing RT-PCR results. Overall sensitivity was 83.3% (95% confidence interval (IC_95_): 78.2–87.4%—Table 2). Taken individually, each assay's sensitivity was not significantly different from the others, ranging from 78.3 to 87.7%. Only the BD Veritor™ was conducted on a sufficient number of patients to allow a meaningful comparison between the emergency room (sensitivity: 88.2%, IC_95_: 76.6–94.5%) and the general practitioners (sensitivity of 87.3%, IC_95_: 76.0–93.7%), yielding no significant difference. Sensitivity according to days since onset of symptoms (DSO), dropped significantly from 86.9% (IC_95_: 81.6–90.8%) for up to 4 DSO to 63.6% (IC_95_: 46.6–77.8%) from 5 DSO (*t*-test, *p* < 0.001). False negative antigen rapid diagnostic tests had C_T_ ranging from 4.93/32 to 29.02/32.

**Table 2:** Compared analytical performances of four SARS-CoV-2 antigen rapid diagnostic tests used in a point-of-care setting at the emergency room of Saint-Pierre University Hospital (Brussels, Belgium) and at a general practitioner consultation.

	**N**	**Sensitivity (IC_**95**_)**	**False negative median C_**T**_ (range)**
Overall	478	83.3% (78.2–87.4%)	17.60 (4.93–29.02)
**Manufacturer**			
BD Veritor™ SARS-CoV-2	177	87.7% (80.1–92.7%)	15.46 (4.93–18.54)
- At the general practitioner consultation	110	87.3% (76.0–93.7%)	
- At the emergency room	67	88.2% (76.6–94.5%)	
Panbio™ COVID-19 Ag Rapid Test Device	101	80.8% (68.1–89.2%)	18.32 (10.29–23.68)
Coris COVID-19 Ag Respi-Strip	135	80.0% (69.2–87.7%)	21.56 (15.52–29.02)
SD Biosensor™ SARS-CoV-2 Rapid Antigen Test	65	78.3% (58.1–90.3%)	15.53 (14.92–16.15)
**DSO**			
<5 DSO	395	86.9%^*^ (81.6–90.8%)	18.38 (10.90–29.02)
- 0–1 DSO	97	89.1% (78.2–94.9%)	
- 2 DSO	118	90.3% (80.5–95.5%)	
- 3 DSO	118	80.3% (68.7–88.4%)	
- 4 DSO	62	89.3% (72.8–96.3%)	
≥5 DSO	53	63.6%^*^ (46.6–77.8%)	15.46 (4.93–27.02)

*DSO, days since symptoms onset; N, number of performed tests; IC_95_, 95% confidence interval*.

* *p-value < 0.001 (Student's t-test)*.

The user-friendliness was satisfactory for all four antigen rapid diagnostic tests tested (Table 3). The Coris COVID-19 Ag Respi-strip had a less satisfactory rating. The main practical issue was its readiness: its “strip-in-a-tube” format was considered by operators as non-practical and leading to a potential biosafety hazard when the reading is difficult. Notably, SD Biosensor™ and Coris BioConcept did not provide any internal control in their kit. BD Veritor™ was the only kit offering nasal swabbing and automated reading.

**Table 3:** User friendliness assessment of four COVID-19 antigen rapid diagnostic tests, adapted from SKUP/2018/114 protocol.

	**Mean of** ***N*** **= 3 questioned operators**			
**Operation facilities**	**BD Veritor™ SARS-CoV-2**	**Coris COVID-19 Ag Respi-strip**	**Panbio™ COVID-19 Ag rapid test device**	**SD Biosensor™ SARS-CoV-2 Rapid Antigen Test**
To prepare the test	Intermediate (1S 2I)^a^	Intermediate^b^	Intermediate (1S 2I)^b^	Satisfactory (2S 1I)^a^
To prepare the sample	Satisfactory	Satisfactory	Satisfactory	Satisfactory
Application of specimen	Satisfactory	Satisfactory	Satisfactory	Satisfactory
Number of procedure step	Satisfactory	Satisfactory	Satisfactory	Satisfactory
Test design	Satisfactory	Unsatisfactory (2U, 1I)^c^	Satisfactory	Satisfactory
Reading of the result	Satisfactory	Difficult^d^	Satisfactory	Satisfactory
Sources of errors	Satisfactory	Intermediate^d^	Satisfactory	Satisfactory
Hygiene when using the test	Satisfactory	Unsatisfactory^e^	Satisfactory	Satisfactory
Size and weight of the package	Satisfactory (2S 1I)^f^	Satisfactory	Satisfactory	Satisfactory
Storage conditions for tests, unopened package^*^	15–30°C	15–30°C	15–30°C	15–30°C
Storage conditions for tests, opened package^*^	15–30°C	15–30°C	15–30°C	15–30°C
Environmental aspects: waste handling^*^	Special precautions	Special precautions	Special precautions	Special precautions
Intended users^*^	Health care personnel	Health care personnel	Health care personnel	Health care personnel
**Information in instruction in the insert**				
Preparations/Pre-analytic procedure	Satisfactory	Satisfactory	Satisfactory	Satisfactory
Specimen collection	Satisfactory	Intermediate^g^	Satisfactory	Satisfactory
Measurement procedure	Satisfactory	Satisfactory	Satisfactory	Satisfactory
Reading of result	Satisfactory	Satisfactory	Satisfactory	Satisfactory
Description of the sources of error	Satisfactory	Satisfactory	Satisfactory	Satisfactory
Help for troubleshooting	Satisfactory	Satisfactory	Satisfactory	Satisfactory
Readability/clarity of presentation	Satisfactory	Intermediate^h^	Satisfactory	Satisfactory (1I 2S)^j^
General impression	Satisfactory	Intermediate^i^	Satisfactory	Satisfactory (1I 2S)^j^
Measurement principle^*^	Satisfactory	Satisfactory	Satisfactory	Satisfactory
Available insert in ENG + FR + NL^*^	Partly^k^	Partly^k^	Partly^l^	Partly^k^
Time factors^*^				
Required training time	<2 h	<2 h	<2 h	<2 h
Duration of preparations/Pre-analytical time	<6 min	<6 min	<6 min	<6 min
Duration of analysis	10–20 min	>20 min	10–20 min	10–20 min
Stability of test, unopened package	>5 months	>5 months	>5 months	>5 months
Stability of test, opened package	>30 days or disposable	>30 days or disposable	>30 days or disposable	>30 days or disposable
Stability of quality control material unopened	>5 months	No QC provided	>5 months	No QC provided
Analytical quality control^*^				
Reading of the internal quality control	Satisfactory	Unsatisfactory^m^	Satisfactory	Unsatisfactory^m^
Usefulness of the internal quality control	Satisfactory	Unsatisfactory^m^	Satisfactory	Unsatisfactory^m^

* *Objective informational items were filled by the principal investigator*.

a *Caps of the buffer tubes difficult to manipulate*.

b *Requires a tube rack*.

c *The use of a strip in a closed tube with a very difficult capping was not considered practical for the operators*.

d *Difficult reading through a closed tube although transparent*.

e *Operators were forced to open the tubes to extract the strip in case of a doubt with the reading causing biosafety concern*.

f *Oversized packaging compared to the number of test*.

g *Lack of precise instruction*.

h *Lack of clarity*.

i *A quick reference guide would have been appreciated*.

j *Small typo and dense content*.

k *Only available in English*.

l *Not available in Dutch*.

m *Not provided*.

#### Automated Antigen Dosing Assay

Two hundred seventy-nine patients (including 93 asymptomatic patients screened for a scheduled hospitalization) were tested. Their SARS-CoV-2 carriage status was categorized as “unlikely” if dosing below 1.32 pg/mL (*n* = 219, 78.5%), “possible” if dosing from 1.32 to 20.27 pg/mL (*n* = 46, 16.5%) and “certain” if dosing higher than 20.27 pg/mL (*n* = 14, 5.0%). All patients with “certain” results had a positive RT-PCR. Seven patients out of 46 (15.2%) with a “possible” result and five out of 219 (2.3%) with an “unlikely” result were tested positive according to RT-PCR, respectively (Table 4). Thus, the overall sensitivity for asymptomatic patients was of 86.7% (13/15). Hence, using this assay for the pre-admission screening of these 93 patients would have spared 87 RT-PCR (93.5%) for the cost of one missed low-positive (C_T_ = 26.04/32).

**Table 4:** Analytical performances of the Lumipulse® *G* SARS-CoV-2 Ag on target populations in the detection of SARS-CoV-2 using a categorization of the risk system.

	**Lumipulse® automated antigen detection**										
		**Certain (>20.27 pg/mL)**		**Possible (1.32–20.27pg/mL)**				**Unlikely (≤1.32pg/mL)**			
**PCR result**	**N**	**Positive**	**Lowest C_**T**_**	**N**	**Negative**	**Positive**	**Lowest C_**T**_**	**N**	**Negative**	**Positive**	**Lowest C_**T**_**
Overall	279	14	3.89	46	39	7	15.7	219	214	5	22.61
Scheduled hospitalizations	93	1	12.73	6	4	2	15.7	86	85	1	26.04
Contacts	67	4	6.98	13	12	1	31.23	50	49	1	22.9
Health workers	119	9	3.89	27	23	4	19.92	83	80	3	22.61
- With symptoms	67	8	3.89	13	13	-	-	46	43	3	22.61

## Discussion

In most industrialized countries, the large scale use of RT-PCR to detect active SARS-CoV-2 infections has shown limits in its capacity to broadly screen the population while providing timely and therefore meaningful results for optimized prevention and treatment. To fill this gap, SARS-CoV-2 antigen rapid diagnostic tests and molecular point-of-care tests are now considered as an adjunct to the RT-PCRs performed on large automated platforms (25).

Our results provide substantial evidence that no current antigen rapid diagnostic test is sensitive enough to be performed on UTM specimen (i.e., at the laboratory). During the first wave in Europe, we proposed a strategy combining antigen rapid diagnostic tests and RT-PCR, both performed in the laboratory (26). We stopped using antigen rapid diagnostic tests in the laboratory during the declining phase of the epidemic, not because of their low sensitivity [as stated by colleagues (27)], but because the proportion of samples from recently infected patient dropped, impairing these tests' usefulness (28). Regular follow-up of the positivity rate could allow adaptations of antigen rapid diagnostic test strategy as proposed by CDC (11) and ECDC (13). Here, we demonstrate the added-value of antigen rapid diagnostic tests at the point-of-care level for <5-days symptomatic outpatient thanks to their ease-of-use, rapid time-to-result, and low cost.

Our results show slightly lower sensitivity than previously reported (25). Indeed, part of the false negative results observed is likely due to variability in the adherence to protocol regarding sampling, incubation time and DSO. Sensitivity and specificity of such antigen rapid diagnostic tests strongly depend on their good execution and reading which are harder to achieve at the frontline where the expertise of personnel can vary; especially in this time of pandemic when the turn-over is higher than usual. This was confirmed by other recently published studies targeting the same population, with sensitivity ranging from 70.0 to 80.4% (29–31).

**Figure 3:**
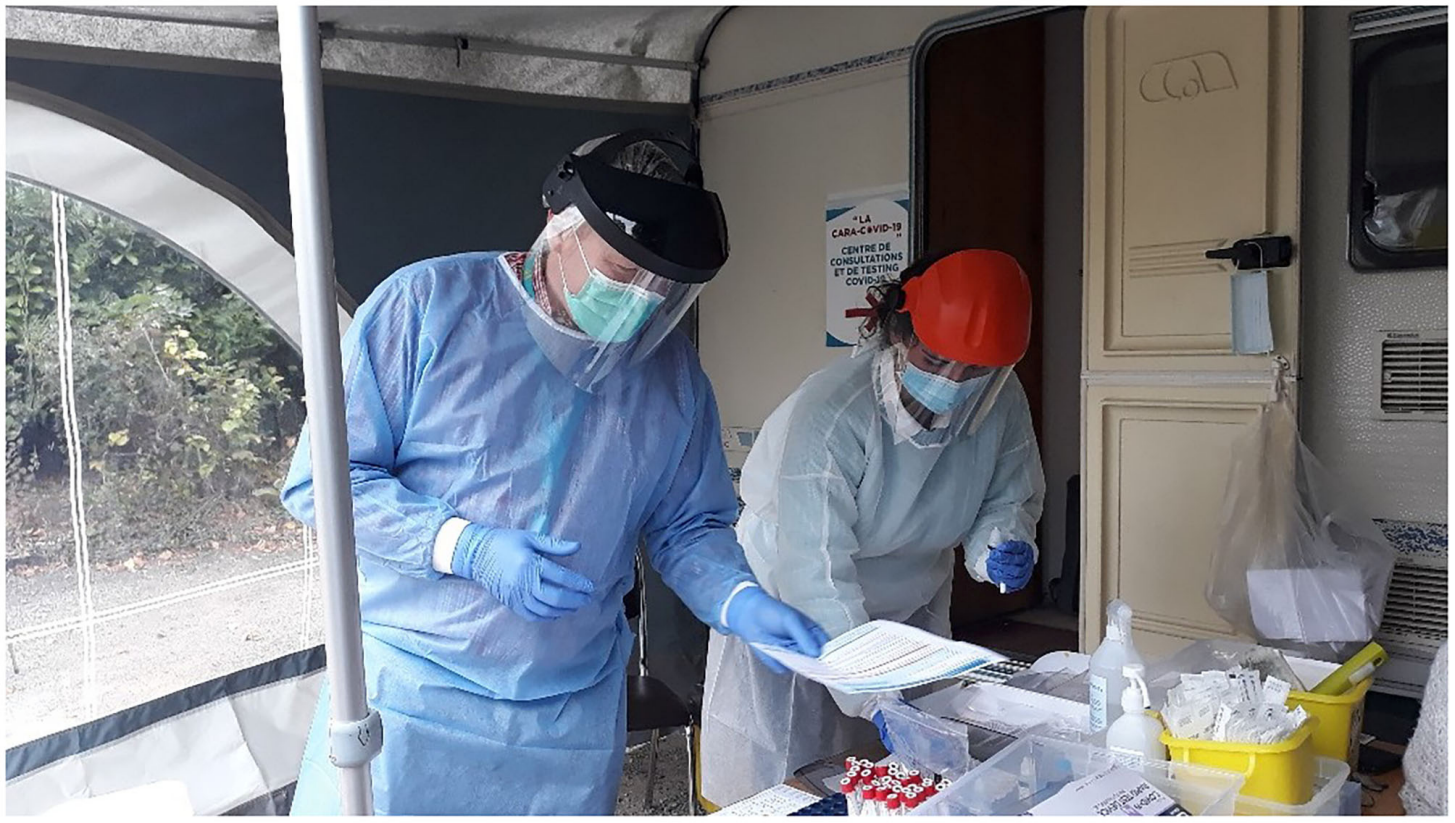
Diagnostic center set outside under a tent by a general practitioners group in Uccle, Belgium (October 22, 2020).

The absence of significant difference between antigen rapid diagnostic tests clinical performances highlights the need to assess their user-friendliness as a main criterion of choice. Our analysis underlined the need to consider very practical aspects such as opening caps while wearing gloves, ensuring biosafety outside a laboratory (see Figure 3) and instructions targeting non-laboratory operators, as recently discussed for low-resource settings (32). Besides, an immediate, in-person communication of a positive result likely allowed a stronger message and a better adhesion regarding quarantine, hygiene and contact-tracing than if done through virtual means, days after the consultation.

The Cobas® Liat yielded stunning performances for a 20-min triplex molecular point-of-care test compared to our RT-PCR. However, invalid results were experienced with viscous samples. The addition of a molecular point-of-care test for patients attending the emergency room and needing hospitalization, regardless of the suspicion of COVID-19, allowed a faster management of inpatients avoiding the admission of asymptomatic SARS-CoV-2 carrier in “COVID-free” units, or the admission of SARS-CoV-2-negative patients in COVID-19 units pending their RT-PCR results. Furthermore, influenza and SARS-CoV-2 co-detection allows a better surveillance at a time where the potential co-circulation of the influenza and SARS-CoV-2 is still unknown. The costs of these molecular point-of-care tests stay high and their availability low. Hence, their use should be considered by targeting the best population with regards to the reduction of global costs related to isolation, use of protective equipment and prevention of nosocomial clusters.

In the present study, the Lumipulse® *G* SARS-CoV-2 Ag showed an overall good analytical performance compared to RT-PCR; and more specifically, to exclude negative and low positive samples using different criteria and cut-off values than the ones proposed by the manufacturer. These cut-offs need to be adapted and chosen regarding the local epidemiology and the objectives of the screening. Our cut-off values diverged from the one proposed in a previous study (17). However, despite the fact we added a viral deactivation by heating, our results yielded a better AUC of the ROC curve. In case of limited access to RT-PCR, such technique can allow testing people who would be otherwise not tested. Its higher throughput and sensitivity than antigen rapid diagnostic tests and its faster time-to-result than RT-PCR make it an interesting intermediary tool. Its low costs and its probable good assessment of infectiousness allow a relevant periodic testing in terms of infection control. Therefore, using antigen dosing could be the best solution to repeatedly test high number of high risk contacts while sparing RT-PCR resources. However, their biosafety must be carefully considered and viral neutralization applied if needed; viscous samples may cause pipetting errors and specific interpretation algorithm should be elaborated.

Our study presents some limitations. We did not consider alternative specimens for SARS-CoV-2 detection such as saliva, the use of serology or broad molecular “syndromic” respiratory panels that could be of use in a larger diagnostic algorithms (33). The emergence of new variants should not impact the value of our algorithm due to the different targets of the assays. However a careful follow-up of their performances over time should be implemented.

## Conclusion

In conclusion, our study underlines the importance of shifting our attention from a narrow focus on the sole analytical performances of the diagnostic tools available (especially when these are similar) to an integrated approach taking into account (i) practical consideration such as time-to-result, field ease-of-use, availability of reagents (ii) target populations (iii) intended use of produced results, and (iv) kinetic of the epidemic. Hence, we elaborated here a diagnostic algorithm based on these considerations to optimize the use of the newly extended arsenal of SARS-CoV-2 direct diagnostic tools, from the decentralized setting to the automated lab, to ensure clinical microbiologists enough ammunition for a reliable and meaningful COVID-19 diagnostic.

## Data Availability Statement

The raw data supporting the conclusions of this article will be made available by the authors, without undue reservation.

## Ethics Statement

Ethical review and approval was not required for the study on human participants in accordance with the local legislation and institutional requirements. Written informed consent from the participants' legal guardian/next of kin was not required to participate in this study in accordance with the national legislation and the institutional requirements. Written informed consent was obtained from the individual(s) for the publication of any potentially identifiable images or data included in this article.

## Author Contributions

NY, CDe, MD, F-ZB, FP, MW, and CDu did the investigations. NY and MH contributed to literature review and the writing of the initial draft. NY, CM, FP, HD, ND, M-LD, and SM contributed to manuscript revision, data compilation, and figure presentation. All authors provided critical review and commentary. NY, ND, FC, MB, and MH contributed to study design, manuscript preparation, literature review, revision, and project administration. All authors contributed to the article and approved the submitted version.

## Conflict of Interest

Becton Dickinson, Coris BioConcept, and Fujirebio have offered reagents for this study. The authors declare that the research was conducted in the absence of any commercial or financial relationships that could be construed as a potential conflict of interest.

## Footnotes

^1^Bundesministerium für Soziales Gesundheit Pflege und Konsumentenschutz. Österreichische Teststrategie SARS-CoV-2.

^2^Robert Koch Institut. Hinweise zur Testung von Patienten auf Infektion mit dem neuartigen Coronavirus SARS-CoV-2.
