# Supplementary material for: SARS-CoV-2 Diagnostic Tests: Algorithm and Field Evaluation From the Near Patient Testing to the Automated Diagnostic Platform
Source: Front Med (Lausanne). 2021 Apr 6;8:650581. doi: 10.3389/fmed.2021.650581 (PMC8055843; doi:10.3389/fmed.2021.650581)

Supplementary Figure S2: Receiver operating characteristic (ROC) curve analysis of the Lumipulse® *G* SARS-CoV-2 Ag when compared to a cycle threshold (C_T) < 20/32. The test achieved an area under the ROC curve (AUC) value of 0.984 ± 0.007.

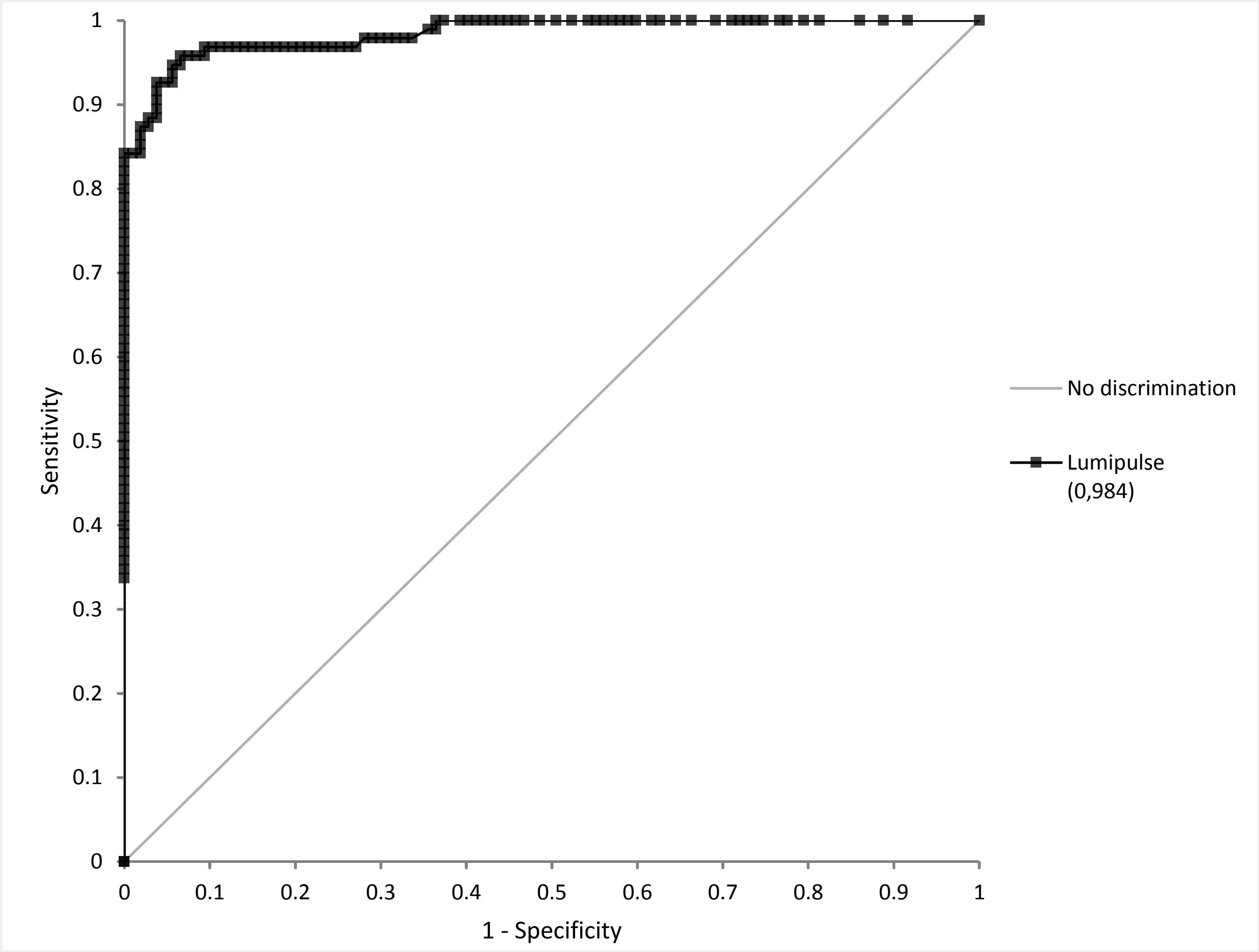

Supplement: Supplementary file 2 [file Image_2.PDF]
