# Supplementary material for: SARS-CoV-2 Diagnostic Tests: Algorithm and Field Evaluation From the Near Patient Testing to the Automated Diagnostic Platform
Source: Front Med (Lausanne). 2021 Apr 6;8:650581. doi: 10.3389/fmed.2021.650581 (PMC8055843; doi:10.3389/fmed.2021.650581)
Supplement: Supplementary file 3 [file Table_1.PDF]

Supplementary Table S1: Possible case definition provided by the Belgian national health institute (Sciensano) for COVID-19

Possible case definition	Criteria
Major symptoms	Minimum 1 of the following of acute apparition without other obvious cause:<br>- Cough<br>- Dyspnoea<br>- Chest pain<br>- Anosmia<br>- Dysgeusia
Minor symptoms	Minimum 2 of the following without other obvious cause:<br>- Fever<br>- Muscle pain<br>- Asthenia<br>- Rhinitis<br>- Sore throat<br>- Headache<br>- Anorexia<br>- Aqueous diarrhea<br>- Confusion<br>- Sudden fall
Chronic respiratory symptoms	Aggravation without other obvious cause of any chronic symptom (COPD, asthma, chronic cough…)
