# Supplementary material for: SARS-CoV-2 Diagnostic Tests: Algorithm and Field Evaluation From the Near Patient Testing to the Automated Diagnostic Platform
Source: Front Med (Lausanne). 2021 Apr 6;8:650581. doi: 10.3389/fmed.2021.650581 (PMC8055843; doi:10.3389/fmed.2021.650581)
Supplement: Supplementary file 4 [file Table_2.PDF]

Supplementary Table S2: Summarized instructions for use of four COVID-19 antigen rapid diagnostic tests (Ag-RDTs).

Manufacturer	Sample	Extraction buffer	Type	incubation	Reading
**Panbio™ COVID-19 Ag Rapid Test Device**	Nasopharyngeal dry swab	300µL to add in an extraction tube	Cassette	15 minutes	Visual
**BD Veritor™ SARS-CoV-2**	Nasal dry swab	dispatched in individual tubes	Strip	15 minutes	Automated
**Coris COVID-19 Ag Respi-Strip**	Nasopharyngeal dry swab	200µL to add in an extraction tube	Cassette	30 minutes	Visual
**SD Biosensor SARS-CoV-2 Rapid Antigen Test**	Nasopharyngeal dry swab	dispatched in individual tubes	Cassette	15 minutes	Visual
